# Extending the Lifetime of Clothing through Repair and Repurpose: An Investigation of Barriers and Enablers in UK Citizens

**DOI:** 10.3390/su141710821

**Published:** 2022-08-30

**Authors:** Lisa Zhang, Jo Hale

**Affiliations:** UCL Centre for Behaviour Change, University College London, London WC1E 7HB, UK

**Keywords:** clothing repair and repurpose, sustainable fashion, sustainable consumption, extended use, behaviour change, theoretical domains framework

## Abstract

Repairing and repurposing clothes can extend their lifetime, helping reduce the environmental impacts of the fashion industry. We aimed to investigate influences on clothing repair and repurpose using the Theoretical Domains Framework. A survey was conducted with a representative sample of 300 UK citizens. The frequency of, and influences on, clothing repair and repurpose behaviour were measured with self-report scales and a free-text item. Quantitative (logistic regression) and qualitative (thematic) analyses were used to identify barriers and enablers of behaviour. Findings showed that participants typically engaged in the behaviour every six months. The main barriers concerned a lack of skills, poor product design, unaffordability of repair services, and incongruence with identity. Key enablers concerned the ability to focus during DIY tasks, dynamic social norms, beliefs about benefits of repairing, emotional attachment to clothing, and having routines and habits of repairing. This study is the first to apply the Behaviour Change Wheel to identify intervention types and behaviour change techniques that can modify these influences, such as training workshops and the provision of free repair and repurpose services. Policy options were suggested to support implementation, such as extended producer responsibility. Awareness and skill-building campaigns, while important, are not enough to support behaviour change; structural and policy changes are needed.

## 1 Introduction

The fashion industry is a major contributor to climate change, biodiversity loss, and pollution [1,2]. ‘Fast fashion’, which relies on cheap and quick manufacturing, frequent purchases and short-lived use of clothes, heavily contributes to these problems [3–5]. An urgent transformation is needed from this model to a circular system where resources remain in use for as long as possible [6]. Yet, there has been relatively slow progress in implementing policies that tackle unsustainable fashion practices [7]. To achieve UK policy goals, a new voluntary agreement—Textiles 2030—has been launched by the Waste & Resources Action Programme (WRAP), which aims to accelerate industry action towards circularity. Alongside technological changes within industry, citizen behaviour change at the consumption stage is also necessary [8]. The beginning of the COVID-19 pandemic saw a pronounced decrease in expenditure on clothes [9] and attitude shifts towards sustainable clothing consumption [10]. However, as restrictions have eased, clothing consumption has appeared to return to pre-pandemic levels [11]. Without intervention, trends suggest that clothing purchases will continue to increase, yet worn for shorter periods of time before being prematurely discarded [12].

Citizens can participate in a variety of sustainable fashion behaviours, typically grouped into three phases: (1) acquisition (e.g., buying second-hand) [13]; (2) use and maintenance (e.g., repairing) [14]; and (3) disposal (e.g., donating to charity) [15,16]. To date, much of the literature has focused on the acquisition and disposal phases [17]. Limited research has investigated the use and maintenance phase, which is essential to extending clothing lifetimes [18]. Part of this phase is ‘repair and repurpose’, which encompasses four related behaviours [19,20]: repairing, where a faulty or damaged clothing product is restored to a functional state (e.g., sewing a loose button on a blouse) [4]; altering, defined as adjusting the fit of a clothing product (e.g., hemming trousers) [21]; upcycling, which involves transforming unwanted clothes into something aesthetically valuable (e.g., turning trousers into shorts) [21]; and repurposing, which changes a garment’s form to a new use area (e.g., turning a shirt into a pillowcase) [20]. Repairing and altering can extend a garment’s technical lifetime (amount of time the item functions as intended), while upcycling and repurposing can prolong its aesthetic lifetime (amount of time one finds the item attractive), although both require similar skills and the motivations for doing them coincide [20].

Repair and repurpose behaviour is not currently common in most Western societies. Diddi and Yan [14] found 55% of US participants never or rarely repaired their clothes. Several initiatives have been introduced to encourage clothing repair and repurpose. In the UK, the ‘Love Your Clothes’ campaign by WRAP provides online guides and training videos. Few retailers currently offer free repair services, and there have been calls to make these services widespread given the high interest from citizens [22]. Some European countries have introduced fiscal policies by reducing the value-added tax on repair services [5]. To make repair and repurpose more widespread, we can consider this as a behavioural problem and apply tools from behavioural science. This firstly involves diagnosing the reasons for current behaviour. The most common barriers to clothing repair and repurpose are a lack of time, equipment and skills, and the high costs involved [14,18,23]. Middleton [24] contends these barriers are relatively easy to address compared to beliefs that there is no need to repair when fast fashion is so widely available [25], and that it is laborious, effortful, and may not be successful [21,26,27]. Contrastingly, awareness and concern about the environmental impacts of clothing, and the belief that repair and repurpose can bring environmental benefits such as less waste, have been identified as encouraging the behaviour [20,22,28]. These motivations are conscious and reflective, but automatic motivational processes are also important. These include routines and habits to dispose of damaged and unwanted clothes, as well as positive moods and emotions which can be linked to repairing and to the clothes themselves [29–32]. Social processes also influence repair and repurpose behaviour, which some associate with poverty, ‘women’s work’, and older age [33,34]. There lack social norms to repair and repurpose [19,35] and, instead, people feel social pressure to buy new [36]. Nevertheless, there appears to be a revival of a ‘mending culture’ through community movements (e.g., Repair Cafés, Street Stitching) [14], illustrating how a dynamic norm (shift in a social norm over time) [37] may promote behavioural change.

Despite identifying a range of behavioural influences on repairing and repurposing, there are some gaps in the existing body of literature. Most previous studies have used qualitative designs focusing mainly on a certain demographic—in particular, females or those who already regularly repair and repurpose (e.g., [30]). The extent to which the findings generalise to a representative sample is unclear. Limitations of studies using a quantitative design (e.g., [14]) include the use of proxy measures of behaviour such as intention, though the intention–behaviour gap is well-known in relation to pro-environmental behaviour [38] and more widely [39]. A further limitation is the lack of theory used to guide the investigation of what is driving repair and repurpose (cf. [40]). In the few studies that applied theory, they were restricted to social cognition (e.g., Theory of Planned Behaviour), which focus on reflective processes. This limits their utility, since many behaviours in the use and maintenance phase are governed by routines and habits [23]. A related criticism is that such theories are individualistic [8,41]. Rather, structural and environmental factors can outweigh individual factors in predicting material consumption [42].

**Figure 1:**
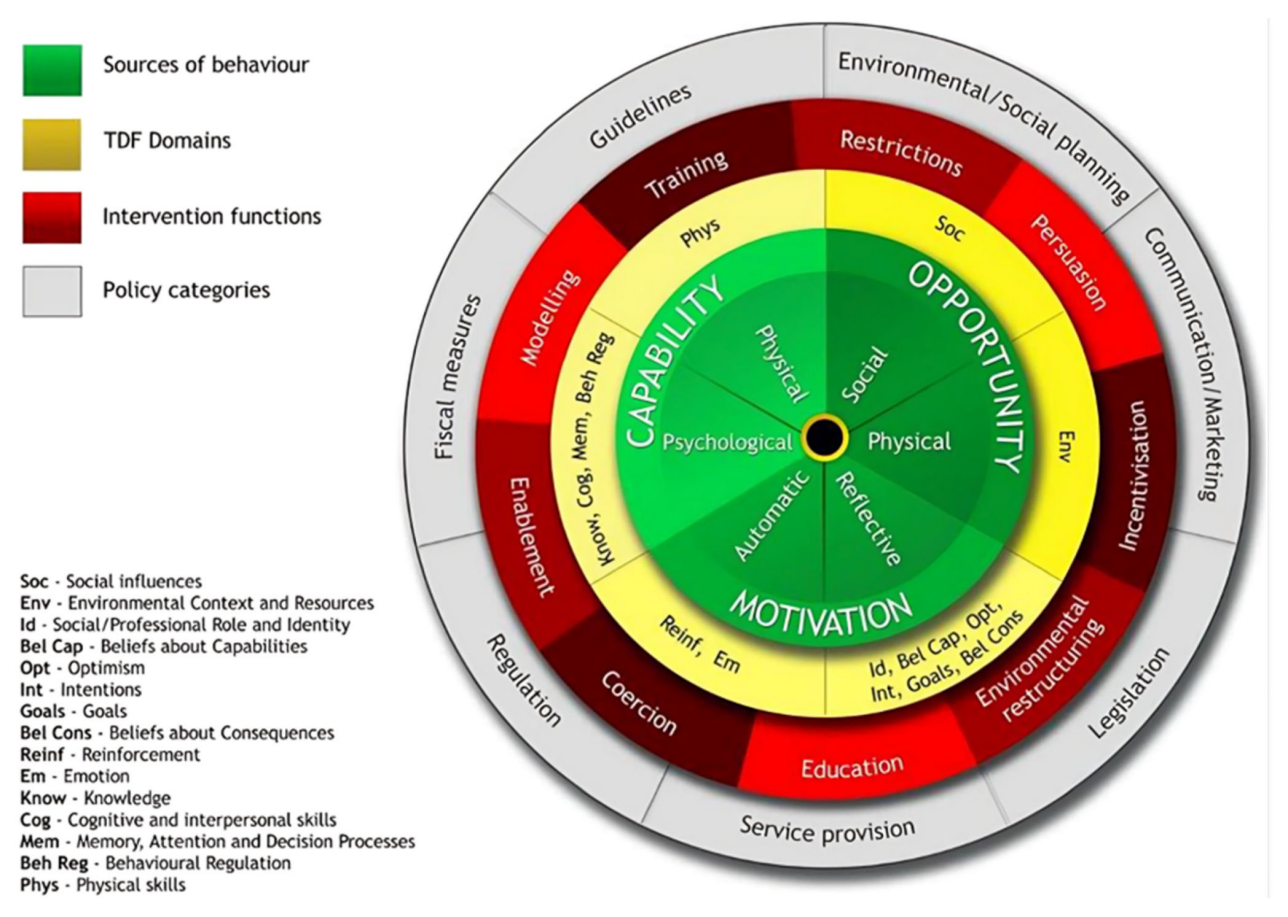
The Behaviour Change Wheel and Theoretical Domains Framework.

A thorough assessment of the target behaviour and its influences provides a robust basis for intervention development. To date, no interventions have been systematically developed to facilitate clothing repair and repurpose. In the present study, we address this gap by applying the Behaviour Change Wheel (BCW; see Figure 1), a synthesis of nineteen frameworks of behaviour change interventions [43]. At the hub of the wheel is the Capability Opportunity Motivation–Behaviour (COM-B) model, which can be elaborated into 14 more detailed domains described in the Theoretical Domains Framework (TDF) [44]. The TDF is an integrative framework that synthesises affective, cognitive, social, and environmental constructs used in theories of behaviour and behaviour change [45]. An analysis of the TDF domains influencing a target behaviour can be used to guide the selection of intervention types most likely to achieve behavioural change. This then guides the choice of policy options, which support delivery of the intervention types. Additionally, the intervention types are linked to a taxonomy of observable behaviour change techniques (BCTs) [46] which specify the intervention content. The BCW has been extensively applied in a range of contexts, particularly implementation science and health, but less so in environmental sustainability.

This exploratory study aimed to answer three research questions:

1. What is the current behaviour with respect to clothing repair and repurpose among UK citizens?
2. Using the TDF, what are the main barriers and enablers to clothing repair and repurpose in UK citizens?
3. Using the BCW approach, what intervention types, policy options, and behaviour change techniques can facilitate clothing repair and repurpose in UK citizens?

These questions were addressed through a survey with quantitative and qualitative components, administered to a representative sample of UK citizens. This study is the first to apply the TDF and BCW within the sustainable fashion literature to understand the target behaviour and design a behaviour change intervention.

## 2 Materials and Methods

### 2.1 Design

This was an observational, cross-sectional study consisting of an online survey. This study was pre-registered on the Open Science Framework (OSF) at https://osf.io/3npzh (accessed on 25 May 2021).

### 2.2 Participants

Participants were recruited using the Prolific online recruitment platform (https://www.prolific.co/ accessed on 16 June 2021), with the inclusion criteria of being aged over 18 years and currently residing in the UK. The recruited sample consisted of 300 participants, nationally representative based on age, sex, and ethnicity. A nationally representative sample was chosen to maximise the generalisability of the results and their relevance to identifying policy options. This sample size was deemed sufficient for a multivariate logistic regression analysis with 14 independent variables (TDF domains) to address research question 2, based on previous rules of thumb [47].

### 2.3 TDF Scale Development

Having identified no pre-existing validated scale to measure influences on clothing and repair and repurpose, a new scale was developed using the TDF. This involved reviewing previous research on influences on the target behaviour, which were categorised according to TDF domains. Validated TDF scales of other behaviours were also used to inform item development (e.g., [48,49]). An initial set of 66 items were developed by author LZ and reviewed by author JH to ensure face validity. Based on feedback, items were modified (e.g., re-wording, shortening) and reduced to 40 (see Table 1 for examples and Supplementary Materials for full scale). The scale was piloted to ensure interpretability and estimate completion time. Items were positively and negatively worded to counter acquiescence bias. Formal scale validation was not conducted due to time constraints.

### 2.4 Survey Measures

1. Demographics. Participants supplied their age, gender, ethnicity, and UK region of residence based on Census 2021 [51].
2. Clothing Repair and Repurpose Current Behaviour. Participants responded to two items. The first item asked about their current behaviour: “During COVID-19, over the past year, how often did you repair and repurpose your existing clothes, instead of disposing of them and buying new clothes?” Participants were prompted to think about the period from March 2020 to June 2021. The second item asked: “Before COVID-19, over the course of a typical year, how often did you repair and repurpose your existing clothes, instead of disposing of them and buying new clothes?” Participants were prompted to think about before restrictions imposed in March 2020. Response options for both items were: (1) Never, (2) About once a year, (3) About once every six months, (4) About once a month, and (5) About once a week.
3. Clothing Repair and Repurpose Intentions. One item asked what participants intended to do after COVID-19 restrictions were removed (“In the future, I intend to repair and repurpose my existing clothes, instead of disposing of them and buying new clothes…”). Response options were: (1) a lot less than before COVID-19, (2) a little less than before COVID-19, (3) about the same as before COVID-19, (4) a little more than before COVID-19, or (5) a lot more than before COVID-19.
4. Influences on Clothing Repair and Repurpose Behaviour. Participants responded to the 40-item TDF scale using a 5-point Likert scale from (1) Strongly disagree to (5) Strongly agree. In addition, we asked a free-text item: “Please tell us your reasons for why you do or do not repair and repurpose your clothes”. This item was included for the purposes of data triangulation (consistent findings across multiple approaches may increase validity) [52] and to identify factors important to participants that may not fit within TDF domains [53].
5. Clothing Repair and Repurpose Tasks (adapted from [12,26]). This section asked participants how likely they would repair or repurpose clothing in different ways. A total of 10 items were presented, such as sewing on a button, and adjusting sizing. Participants responded using a 5-point Likert scale from (1) Extremely unlikely to (5) Extremely likely.

### 2.5 Procedures

All subjects gave their informed consent for inclusion before they participated in the study. The protocol was approved by University College London Research Ethics Committee (approval number CEHP/2020/579). The survey was administered online using Qualtrics software. Participants read a pre-amble which defined repair and repurpose, then completed the above measures. Four attention checks were included to identify non-serious attempts. The study took under 10 min to complete. Participants were compensated for their time (£1.00, or £7.89/h).

### 2.6 Data Analysis

Missing data, potential outliers, and non-serious attempts were checked by examining boxplots, histograms, and scatterplots for the variables of interest.

To answer research question 1 (what is the current behaviour with respect to clothing repair and repurpose?) we examined frequencies of clothing repair and repurpose current behaviour, future intentions, and clothing repair and repurpose tasks (see above survey measures).

To address research question 2 (what are the main barriers and enablers to clothing repair and repurpose?), items from the TDF scale were scored positively (negatively worded items reverse coded), summed, and a mean score for each TDF domain was computed, consistent with previous research [54]. Internal consistency of each domain was assessed using Cronbach’s alpha; a cut-off of 0.5 was considered satisfactory for preliminary research using TDF scales [55,56], and given this study was exploratory in nature. Mean TDF domain scores were used in two ways to assess which domains acted as barriers and enablers. Firstly, a higher mean score was taken to indicate a stronger enabler, and a lower mean score as a stronger barrier [54]. Secondly, ordinal logistic regression analysis was conducted to identify which of the 14 TDF domain scores (independent variables) predicted repair and repurpose behaviour (current behaviour, i.e., during COVID-19). Free-text responses were analysed using an inductive, followed by deductive, approach recommended by McGowan et al. [53]. Themes were generated inductively and then TDF was used as a deductive framework to organise the themes. If multiple themes were generated, to prioritise the most important ones, the following criteria were used based on past research [57,58]: high frequencies (i.e., many participants identified a particular influence); and conflicting beliefs (i.e., participants identified opposite beliefs about the same issue). Based on Braun and Clarke [59], Table 2 details the phases undertaken.

To address research question 3 (what intervention types, policy options, and behaviour change techniques can facilitate clothing repair and repurpose?), the identified barriers and enablers were mapped to BCW intervention types and policy options using linkage matrices from Michie et al. [60]. To select BCTs, ‘most frequently used BCTs’ lists from Michie et al. [60] and the Behaviour Change Technique Taxonomy (BCTTv1) [46] were consulted. Our decisions were informed by the affordability, practicability, effectiveness/cost-effectiveness, acceptability, side-effects/safety, and equity (APEASE) criteria, another step within the BCW approach.

## 3 Results

### 3.1 Preliminary Analyses and Demographics

No outliers or missing data points were identified. Three non-serious attempts (failed multiple attention checks, repeated Likert scale responses in succession) were identified and removed from subsequent analyses. The final sample comprised of 297 participants. Their demographic information is shown in Table 3.

### 3.2 What Is the Current Behaviour with Respect to Clothing Repair and Repurpose?

**Figure 2:**
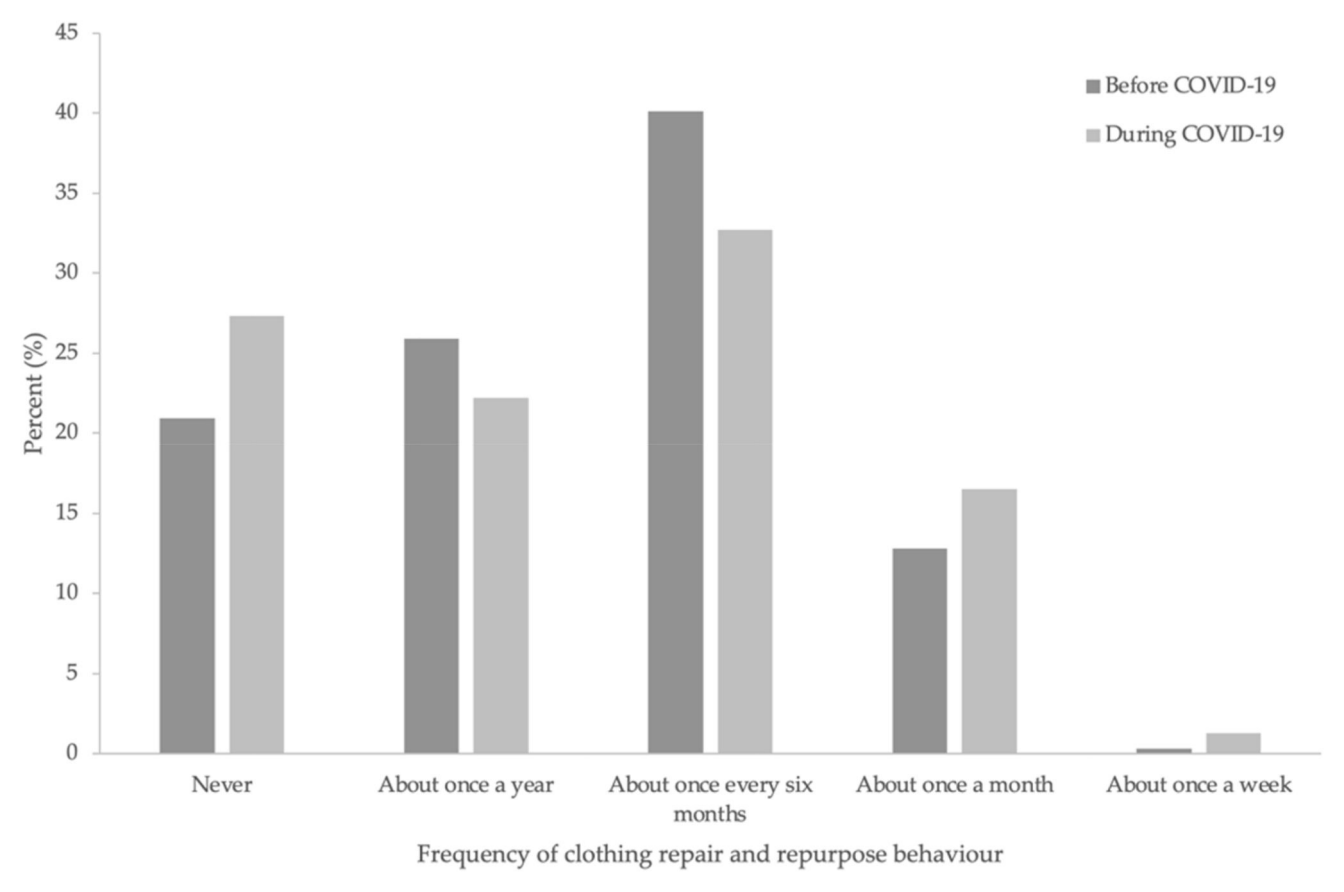
Frequencies of clothing repair and repurpose behaviour before and during COVID-19.

**Figure 3:**
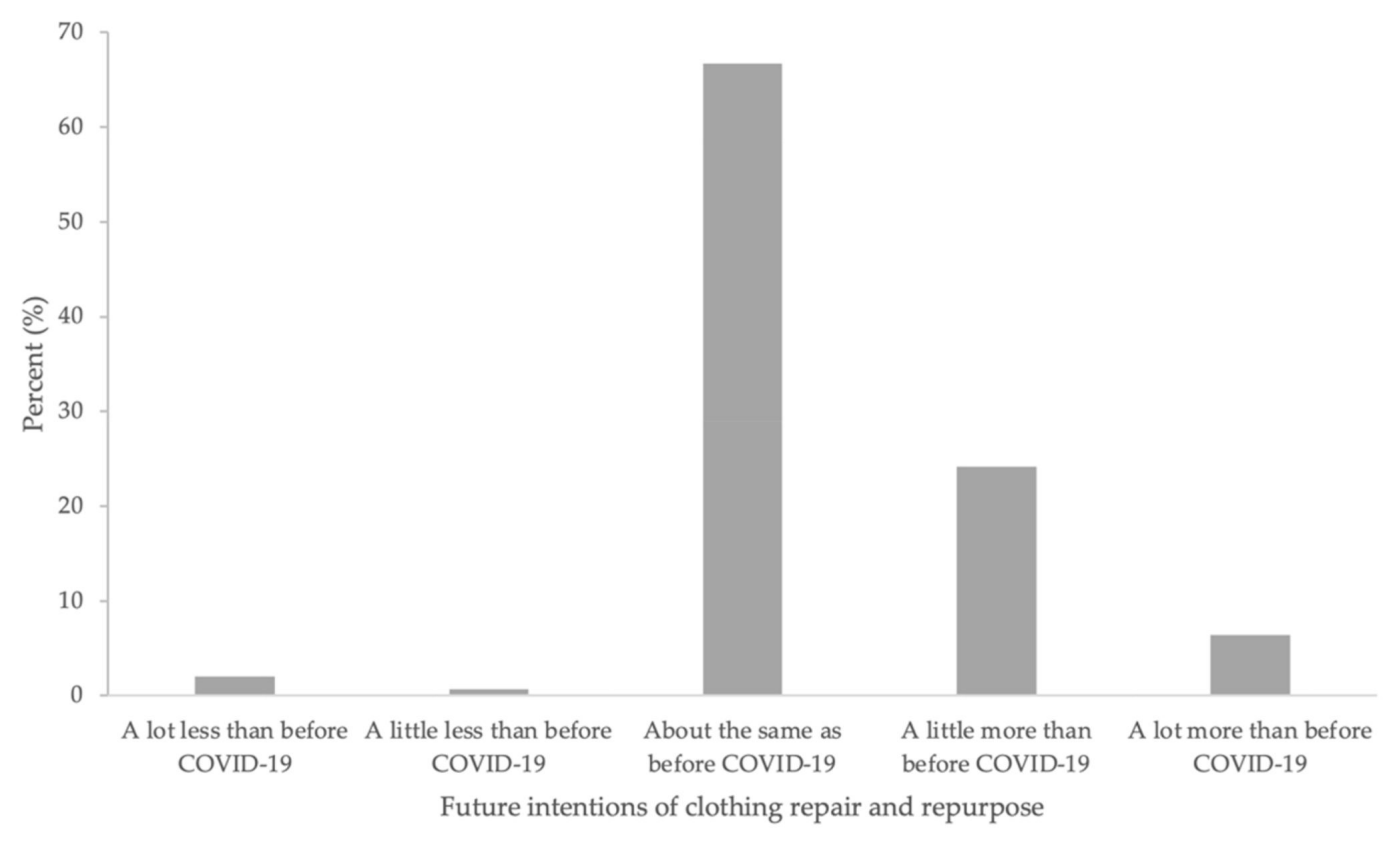
Frequencies of future intentions of clothing repair and repurpose behaviour.

Figure 2 compares frequency histograms of clothing repair and repurpose behaviour before and during COVID-19, whilst Figure 3 presents future intentions. At both timepoints, the most common response was to repair and repurpose clothing once every six months (40.1% and 32.7% before and during COVID-19, respectively). A Wilcoxon signed-rank test showed that frequency of clothing repair and repurpose behaviour did not significantly change from before to during COVID-19 (Z = *−*1.00, *p* = 0.32). The majority (66.7%) indicated that in the future, they intend to engage in the target behaviour about the same as before COVID-19.

**Figure 4:**
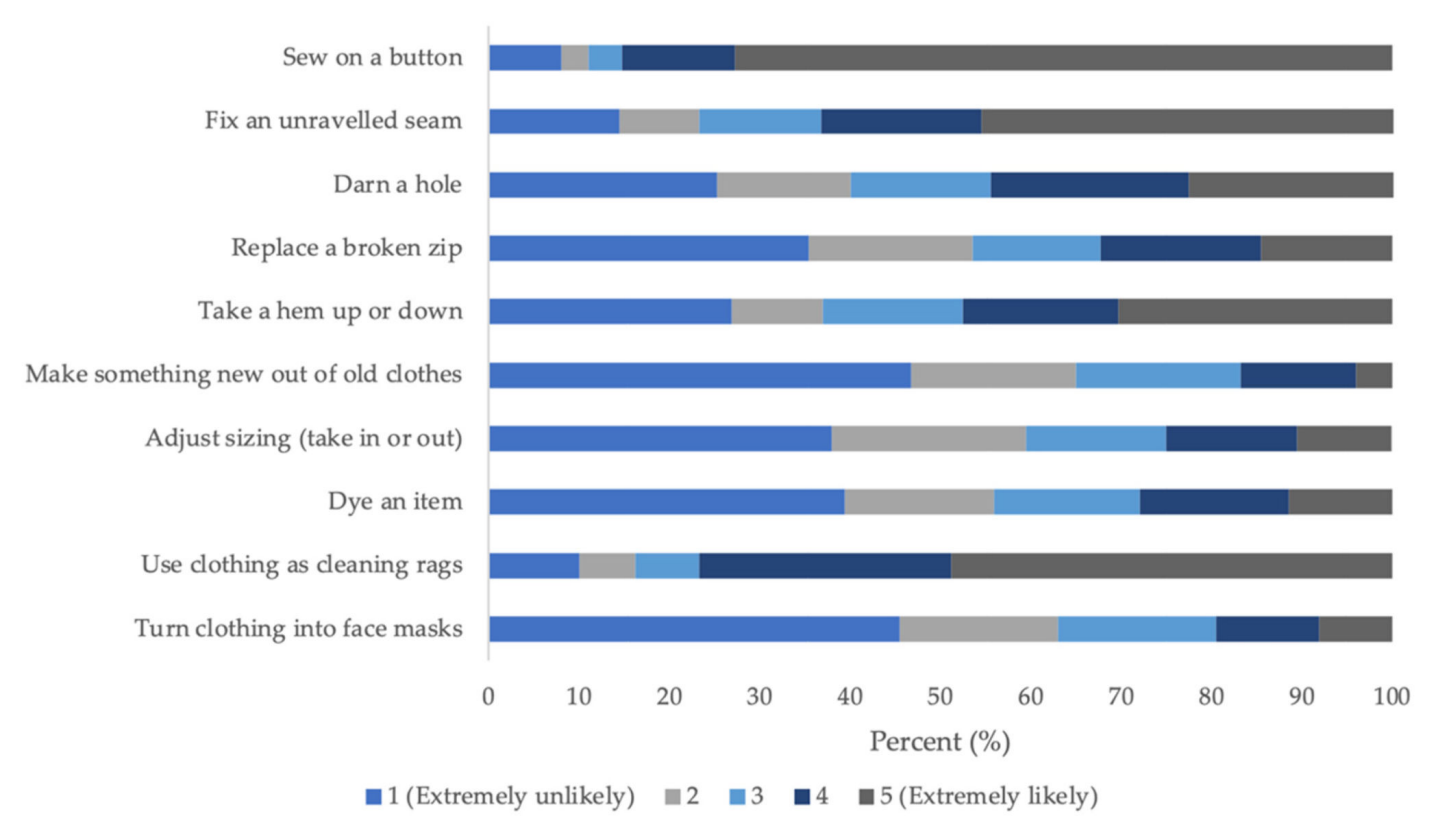
Likelihood of performing clothing repair and repurpose tasks.

Figure 4 shows the likelihood that one would engage in various clothing repair and repurpose tasks. Most people responded that it would be extremely likely for them to perform tasks such as sewing on a button (72.7%), but extremely unlikely to carry out tasks such as making something new out of old clothing (46.8%).

### 3.3 What Are the Main Barriers and Enablers to Clothing Repair and Repurpose?

#### 3.3.1 Descriptive Statistics

When combining responses for each of the TDF domains, four domains had alphas less than 0.5 (knowledge [0.46]; behavioural regulation [0.44]; social influences [0.40]; memory, attention, and decision processes [0.16]). Similar to previous studies [61], these domains were reviewed to determine whether Cronbach’s alpha improved if individual items were removed, and subsequently, three items were excluded from further analysis (one item from each of: social influences; memory, attention, and decision processes; and behavioural regulation). The ‘knowledge’ domain was an exception, as removing items did not improve reliability, indicating heterogeneity within the domain. Results for this domain are still presented; however, interpretations are cautioned.

Descriptive statistics (means, standard deviations) and Cronbach’s alpha for each TDF domain are reported in Table 4. Key enablers (indicated by highest mean scores) were beliefs about consequences and social influences. Key barriers (indicated by lowest mean scores) were identity and skills.

#### 3.3.2 Regression Analysis

To assess which TDF domains are associated with current clothing repair and repurpose behaviour, an ordinal logistic regression was conducted (for completeness, an ordinal logistic regression with repair and repurpose behaviour before COVID-19 as the dependent variable is presented in Supplementary Materials). No multicollinearity was detected between predictor variables (VIF < 10; tolerance > 0.1). However, the assumption of proportional odds was not met, potentially because frequencies were unequally distributed across the response categories for the dependent variable (current behaviour during COVID-19). Given the low frequencies within the ‘About once every week’ category (1.3%, or four participants, recorded this response), it was combined with the ‘About once every month’ category. The proportional odds assumption was tested again and met. The likelihood-ratio test assessing overall model fit was significant, suggesting good fit [62]. McFadden’s pseudo R^2^ was also satisfactory [63].

The results of the regression, including parameter estimates and model fit statistics, are reported in Table 5. The results showed that greater scores on two domains (memory, attention, and decision processes; reinforcement) were associated with significantly higher odds of repair and repurpose behaviour.

#### 3.3.3 Qualitative Analysis

Eighteen themes were generated which reflected different behavioural influences on clothing repair and repurpose behaviour and were categorised according to TDF domains (Table 6). The following domains were prioritised as most important according to our chosen criteria of showing high frequencies and/or conflicting beliefs: skills, environmental context and resources, emotion, and beliefs about consequences.

### 3.4 What Intervention Types, Policy Options, and Behaviour Change Techniques Can Facilitate Clothing Repair and Repurpose?

From the combined quantitative and qualitative behavioural analysis, the following domains were deemed important to change, thus were targeted in intervention development: skills; memory, attention, and decision processes; social influences; environmental context and resources; beliefs about consequences; identity; emotion; and reinforcement.

All nine intervention types were identified to be potentially effective, thus after applying the APEASE criteria (see Supplementary Materials), the six most suitable were: education, persuasion, training, modelling, environmental restructuring, and enablement. Ten BCTs were selected to identify intervention content. Furthermore, all policy options were identified to be potentially effective, and after applying the APEASE criteria (see Supplementary Materials), the four most appropriate were: communication/marketing, fiscal measures, regulation, and service provision. A proposed intervention indicating pathways between the barriers and enablers, intervention types, BCTs, and policy options are shown in Table 7.

## 4 Discussion

Through a representative survey of UK citizens, this research provides novel insights into how often UK citizens repair and repurpose their clothes and the main barriers and enablers to this behaviour. This study is also the first to apply the BCW approach to develop a behaviour change intervention in the context of sustainable clothing consumption.

Most participants indicated they repair and repurpose clothes every six months or less often. This is similar to studies in other Western societies such as Norway, where over half said they had never repaired clothing in the past two years [64], and previous research suggesting the majority of people repaired clothing ‘sometimes’ [26]. Whereas past studies have typically used unspecified time intervals (e.g., never, sometimes, always), which can be ambiguous and lead to inconsistencies within and between participants [65], the present study used an explicit timeframe (i.e., never to once a week). This means our data may be more easily compared across participants and with future research tracking the prevalence of repair and repurpose in the UK or other settings.

The present study used a mixed-methods approach to data triangulation to establish corroborating evidence about influences on repair and repurpose. A lack of skills was identified as a major barrier, as it had the second lowest mean score on the TDF scale, and 23% of the sample indicated it as a barrier in the free-text response. This replicates past research [18]. This is likely due to fast fashion rendering repair and repurpose obsolete, as well as the demise in textile education. On the other hand, the ability to focus one’s attention during DIY tasks was an enabler (memory, attention, and decision processes domain). Whilst increasing skills might be easily addressed, improving attention might prove more difficult to achieve without a longer-term strategy. Introducing textile lessons is one possible solution, as mindful actions such as sewing and designing offer an antidote to fast-paced society [7]. Education and training are best suited to target these influences. Indeed, such intervention types are core components in existing UK behaviour change campaigns, such as ‘Love Your Clothes’ (LYC) by WRAP (https://www.loveyourclothes.org.uk/ accessed on 1 August 2021). The campaign provides free online videos, guides, and workshops (‘Habits for Life’) to equip citizens with skills in repair and repurpose. The findings of this study reiterate the notion that awareness and skill-building campaigns continue to be important components of any intervention for repair and repurpose. Active collaboration is needed from businesses, retailers, and local and national authorities to promote awareness. For instance, the LYC logo could be embedded on clothing labels/tags and retailers’ websites, and pamphlets could be provided with in-store purchases.

The findings show that motivational factors were strong influences. Many people repaired and repurposed for the anticipated benefits (beliefs about consequences domain), which aligns with previous research [20]. People did not consider repairing things to form part of their social identity, which is incongruent with other studies, possibly due to differences in samples. Studies have found strong identities as a ‘mender’ in those who routinely repair and repurpose [66], whereas such an identity did not appear in the present sample, since the behaviour was relatively infrequent. Regarding automatic processes, an emotional connection with one’s clothes suggests that valuing, rather than viewing clothes as disposable, facilitates the behaviour [32]. Conversely, routines of buying new and disposing of old clothes was a strong barrier. What is lacking in existing campaigns is the use of persuasive communication, to target these motivation factors. This research proposes the reframing of repair and repurpose in terms of environmental and economic savings, through compelling language and imagery. Persuasive techniques can foster positive emotions about valuing one’s clothes, and negative feelings about overconsumption and waste.

Multiple barriers regarding one’s environmental context and resources were identified and consistent with past research. This included a lack of time and access to equipment, ease of competing behaviours, financial cost, and poor design features of the garment [50]. This suggests that the fashion industry and government hold key accountabilities in over-coming these barriers through environmental restructuring and enablement intervention types. It is not merely one’s personal responsibility to increase their repair and repurpose behaviour, rather, behavioural change must be embedded in and supported by structural and policy change. Retailers offering free repair and repurpose services and kits hold great effectiveness in helping citizens keep their clothes. It has high interest from a citizen perspective, and can increase brand image and trust [22,67]. From a business side, though, there may be reservations if making a profit is challenging due to increased labour. It is therefore beneficial to include multi-stakeholder perspectives in the fashion system to avoid unintended consequences [35]. For example, a barrier reported by retailers is the short supply of trained personnel in repairs, which might lead to longer wait times and reduced willingness of citizens to use such services [64].

Regulation, service provision, and fiscal policy measures can support the delivery of intervention strategies that target environmental context and resources. ‘Right to repair’ laws have been introduced in the UK, which requires producers to make spare parts available for household appliances. This could be extended to clothing items. Some European countries have introduced tax breaks on repair services, making it more cost-effective than replacing [5]. To encourage retailers to offer free repair and repurpose services, subsidies or tax-cut incentives might be effective [29]. In addition, Extended Producer Responsibility is a regulatory policy in the UK, where producers take responsibility for waste prevention, low-impact product design, and supporting citizen repair and recycling [7]. However, the scheme does not currently include textiles, thus could be reformed to include clothing. The scheme could include durability and repairability disclosures or certifications, which provide information about the expected item lifetime under normal wear and tear, and whether the item is suitable for repair [68]. Citizens can make informed item-comparisons, potentially incentivising brands to improve durability. Such transparency might reduce greenwashing concerns. A step further could be setting minimum design standards for durability and repairability—this might change mindsets that repairing is worthwhile for all clothing.

Lastly, social influences did not present a prominent barrier as it has in past studies (e.g., [33,34]). This might be attributed to the rise in community initiatives and movements, such as Repair Cafes and Street Stitching, which have increased visibility, creating a dynamic norm. To capitalise on this, using role models that citizens identify with may be effective, since clothing is linked to the inspiration of fashion influencers. Similarly, images showing everyday citizens repairing and repurposing in different settings can signal the normality of the behaviour.

The present findings should be interpreted in light of some limitations of the research. The participant sample matched the distribution of the UK population on age, sex, and ethnicity only, but may be less representative of other potentially important demographics, such as socio-economic status. Relatedly, the results may not generalise to other countries, since influences on behaviour likely differ across settings [60]. Due to the time sensitivity of conducting research during the pandemic, the influences on clothing repair and repurpose scale did not undergo formal validation, therefore future research using or adapting this scale would benefit from assessing its psychometric properties, such as factor structure. Further, there were low reliabilities for several TDF domains, possibly due to heterogeneity within a domain. This reflects a limitation with the TDF approach; some domains are broad and can include multiple constructs. For example, items in the environmental context and resources domain relate to access, cost, and clothing quality, and it would be reasonable for participants to respond in different ways. Future research could attempt to break down such heterogenous domains into more well-defined constructs. Finally, there are limitations to the use of self-report in relation to socially desirable responding, such as pro-environmental actions and attitudes (though, as we have noted, some may consider repair and repurpose socially undesirable). Relatedly, Nielsen et al. [69] found that, whilst psychological factors strongly predicted self-reported behaviour of clothing consumption, it weakly predicted clothing greenhouse gas (GHG) emissions. Future research should use objective behaviour measures focused on impact. For example, field observations may give a more objective assessment. Wardrobe studies [70] is a new method which could be used to measure the number and proportion of clothes repaired or repurposed over time (behaviour), as well as how long the items’ lifetimes were extended (outcome), and emissions saved (environmental impact).

## 5 Conclusions

Repairing and repurposing is a powerful way to extend the lifetime of clothing, helping reduce the environmental impacts from the fashion industry. The present research is the first to use two evidence-based behavioural science frameworks, which contributes to the growing body of evidence on sustainable fashion. The identified influences represent targets for an evidence-based intervention to facilitate behavioural change. This can play a pivotal role in achieving policy objectives outlined in recent UK Government blueprints, such as the Waste Prevention Programme for England, in which one of the goals is to “address the negative environmental impacts of the textiles sector and fast fashion” ([71] p. 32). This research reiterates that current awareness and skill-building campaigns, while important, are not enough. Structural and policy changes are also needed to support behavioural change and, ultimately, system change.

## Supplementary Material

Supplementary Materials

## Figures and Tables

**Table 1:** Example Items for TDF Domains.

THF Domain	No. Items	Example Item	Source
Knowledge	3	I know how to repair and repurpose my clothes.	Adapted from Diddi and Yan [14]
Skills	3	I have the physical skills to repair and repurpose my clothes (e.g., dexterity to thread a needle).	Adapted from Huijg et al. [48]
Memory, Attention and Decision Processes	2	I can focus my attention in order to repair and repurpose my clothes.	Content based on Twigger Holroyd [27]
Behavioural Regulation	2	I often put off repairing and repurposing my clothes (e.g., not bothered). *	Content based on EAC [7]
Social Influences	4	I do not repair and repurpose my clothes because other people see it negatively. *	Content based on Gwilt [33]
Environmental Context and Resources	8	I have the necessary equipment (e.g., sewing machine) to repair and repurpose my clothes.	Content based on Fisher et al. [50]
Social/Professional Role and Identity	2	Repairing and repurposing things is part of my identity.	Content based on Lapolla and Sanders [31]
Beliefs about Capabilities	1	I am confident in my abilities to repair and repurpose my clothes.	Adapted from Diddi and Yan [14]
Optimism	1	I am optimistic that the end result of repairing and repurposing my clothes will be successful.	Adapted from Diddi and Yan [14]
Beliefs about Consequences	2	I believe that repairing and repurposing my clothes has positive impacts on the environment (e.g., less waste).	Adapted from Diddi and Yan [14]
Intentions	1	I strongly intend to repair and repurpose my clothes.	Adapted from Diddi and Yan [14]
Goals	2	A goal of mine is to learn new skills to repair and repurpose my clothes.	Adapted from Bhatt et al. [28]
Reinforcement	4	I routinely dispose of my clothes instead of repairing and repurposing them. *	Content based on Goworek et al. [23]
Emotion	5	I repair and repurpose my clothes because I have emotional attachment to them.	Adapted from Diddi and Yan [14]

* Indicates reverse-coded items.

**Table 2:** Phases Undertaken to Analyse Free-text Responses.

Phase	Steps
Phase 1: Familiarisation with data	Reading and re-reading the responses Noting down initial ideas and recurring patterns Coding raw data with labels Developing a codebook where codes are labelled, defined, example quotes given
Phase 2: Generating initial codes	Applying the codebook to revise code labels and definitions
Phase 3: Generating initial themes	Organising codes into potential themes. This includes relationships between codes and between themes Cyclical process of checking if potential themes fit in relation to the code labels and the raw data, re-coding if needed
Phase 4: Reviewing themes	Revising if themes need to be combined, separated, or removed Generating a thematic map of the analysis
Phase 5: Defining and naming themes	Finalising names, definitions, descriptions, and example quotes for each theme
Phase 6: Applying the theoretical framework	Mapping generated themes onto TDF domains of barriers, enablers, or mixed Themes and their categorisations reviewed by supervisor
Phase 7: Producing the report	Tabulating and writing up the analysis, while relating back to the research question

**Table 3:** Demographic Characteristics of Current Sample.

Demographic	Current Sample
Age (years)	
Mean	44.52
SD	15.83
Range	18–88
Gender	
Female	51.5%
Male	48.1%
Other	0.3%
Ethnicity	
White	76.7%
Asian/British Asian	10.7%
Black/African/Caribbean/Black British	6.3%
Mixed or multiple ethnic groups	3.0%

**Table 4:** Descriptive Statistics and Internal Consistencies for TDF Domains.

THF Domain	M	SD	*α* *
Knowledge	3.42	0.83	0.46
Skills	2.72	1.03	0.70
Memory, Attention, and Decision Processes	3.22	1.40	-
Behavioural Regulation	2.73	1.16	-
Social Influences	4.14	0.77	0.62
Environmental Context and Resources	2.88	0.66	0.63
Social/Professional Role and Identity	2.30	1.11	0.71
Beliefs about Capabilities	2.89	1.39	-
Optimism	3.33	1.23	-
Beliefs about Consequences	3.98	0.83	0.51
Intentions	3.36	1.28	-
Goals	3.02	1.10	0.71
Reinforcement	3.13	0.87	0.64
Emotion	3.19	0.89	0.75

* Cronbach’s Alpha is only presented for domains with at least two items.

**Table 5:** Ordinal Logistic Regression Results Predicting Current Behaviour.

Predictor	*β*	SE (*β*)	Wald’s χ^2^	*df*	*p*	Odds Ratio	[95% CI Odds Ratio]
Knowledge	0.14	0.21	0.46	1	0.50	1.15	[0.77,1.72]
Skills	0.14	0.21	0.47	1	0.49	1.15	[0.77,1.74]
Memory, Attention, and Decision Processes	0.34	0.14	5.80	1	0.02	1.40	[1.07,1.84]
Behavioural Regulation	–0.03	0.11	0.06	1	0.80	0.97	[0.78, 1.21]
Social Influences	–0.19	0.16	1.36	1	0.24	0.83	[0.60, 1.14]
Environmental Context and Resources	0.49	0.29	2.83	1	0.09	1.63	[0.92, 2.89]
Identity	0.06	0.15	0.19	1	0.67	1.07	[0.80, 1.43]
Beliefs about Capabilities	–0.14	0.17	0.67	1	0.41	0.87	[0.63, 1.24]
Optimism	0.07	0.15	0.20	1	0.65	1.07	[0.80, 1.42]
Beliefs about Consequences	0.36	0.21	3.08	1	0.08	1.44	[0.96, 2.15]
Intentions	0.20	0.17	1.30	1	0.25	1.22	[0.87, 1.70]
Goals	0.09	0.15	0.32	1	0.57	1.09	[0.81,1.47]
Reinforcement	0.45	0.23	3.88	1	0.04	1.57	[1.00,2.47]
Emotion	0.10	0.24	0.16	1	0.69	1.10	[0.69, 1.77]
**Test**			χ**^2^**	*df*	***p***		
Test of parallel lines for proportional odds assumption			31.94	28	0.28		
Omnibus test for model fit (Likelihood Ratio)			170.85	14	<0.001		
Pseudo R^2^			0.21 (McFadden R^2^)				

**Table 6:** Descriptions, Frequencies, and Quotes for Generated Themes.

THF Domain	Theme (*n* = 18)	Theme Description	Frequency (%)	Barrier/Enabler/ Mixed	Example Quote(s)	
Knowledge	1 Knowledge of how to repair and repurpose	Knowing how to repair and repurpose, or being aware of services (e.g., tailors) that	18 (6.1%)	Barrier	*"Idontknow how to repair clothes nor do I know someone who can repair clothes"* ID 28	
	2 Awareness of environmental impacts of clothing	Being aware of the impacts of clothing on the environment, which often translated to behaviour	7 (2.4%)	Enabler	*"I know the majority of clothes end up in landfill so I am trying to reduce the amount of clothes that I have ending up there."* ID 87	
Skills	3 Skills and skill development	Having the skills to repair and repurpose, often attributed to (lack of) training, creativity, or physical capability	65 (21.9%)	Barrier	*"I don’t have the skills to repurpose or repair my clothes."* ID 46 *"I never learnt how to repair fabrics"* ID 84	
Social influences	4 Social support	Support from friends and family when one is unable to repair and repurpose themselves	7 (2.4%)	Enabler	*"I usually ask a friend or family member to repair or repurpose an item of my clothing for me if need be."*ID 46
	5 Social feedback and conformity	Receiving negative feedback from others, and conform to fashion trends	10 (3 4%)	Mixed	Barrier: *"there were a few occasions where I received negative feedback when I asked people’s opinions which put me off."* Enabler: *"I am not concerned with items going out of fashion, as I rarely follow fashion trends."* ID 188
Environmental context and resources	6 Accessibility to resources	Access to necessary equipment such as a sewing machine and haberdashery supplies	7 (2.4%)	Mixed	Barrier: *"I lack the equipment needed."* ID 287 Enabler: *"i have a sewing machine in the house where i can used for repair it." ID 233*
	7 Time constraints	Lack of time to carry out repair and repurpose tasks, particularly since they require considerable time	22 (7.4%)	Barrier	*"i dont repair clothes out of having no time to do it"* ID 193 *"Too time consuming to repair or repurpose."* ID 160
	8 Convenience of alternative behaviours	Ease of buying new clothes, and disposing via different routes such as donating, selling, or throwing away	68 (22.9%)	Barrier	*the convenience of just buying new clothes when old ones have had their time appeals to me more."*ID 84 *"I would prefer to hand them on to a more needy cause, or sell them"* ID 178	
	9 Economic viability	One perspective is that clothes are cheaper to replace because repair costs are unaffordable, and new items are inexpensive. Another perspective is that repair and repurpose is more economical than replacing	56 (18.9%)	Mixed	Barrier: *"Most of the clothes I buy cost me less than £10, last a couple of years then I buy again, the cost to repair would be more than buying new"* ID 121 Enabler: *"It makes financial sense to carry out a repair rather than to replace a whole garment."* ID 132	
	10 Characteristics of the item	Characteristics include the item price, material quality and durability, and damage severity (or amount of wear). Expensive, high-quality, durable items, and items with minor damage are more likely to be repaired	89 (30.0%)	Mixed	Barrier: *"Items bought are usually not long lasting and not worth repairing."* ID 119 Enablers: *"If an item is expensive or a quality item, I am prepared to invest time repairing or altering."* ID 149 *“I repair clothes that still has a life in them that only needs a minor repair, i.e small tears etc."* ID 159	
Reinforcement	11 Past experiences	Attaching negative associations to repair and repurpose due to past unsuccessful experiences Repair and repurpose was	3 (1.0%)	Barrier	*“tried and end up ruining some item beyond usable"* ID 148	
	12 Routines	regarded as something one routinely engages in	(2.0%)	Enabler	*It s something I ve always done for years."* ID 213	
Emotions	13 Attachment to clothes and emotions derived	Having an emotional connection with their clothes, valuing them, and enjoying sewing, versus having no attachment and treating clothes as disposable	57 (19.2%)	Mixed	Barrier: *"More common that I go off an item of clothing so discard it."* ID 181 Enablers: *"I care about and tend to get attached to objects, so I try to keep a hold of them for as long as possible. This makes it very likely for me to repair something I own."*ID 34	
Beliefs about capabilities	14 Self-efficacy	Confidence in one’s skills, which is moderated by the perceived difficulty or ease of the task	31 (10.4%)	Mixed	Barrier: *"I’m not confident enough to do this myself."* ID 71 Enabler: *"I am quite good at sewing, so have the confidence to do this."* ID 249	
Beliefs about consequences	15 Anticipated consequences (outcome expectancies)	Beliefs that repair and repurpose has positive impacts on the environment, get one’s money’s worth, and can improve clothing functionality, aesthetics, and uniqueness	82 (27.6%)	Mixed	Barrier: *"I would also feel that even if it was professionally restored that it will not be as good as it used to be."* ID 22 Enabler: *"Repairing an item can extend it’s useful life and represents good value for the time taken."* ID 80	
	16 Attitudes	Having a dislike of repaired clothes and seeing it is unworthwhile, versus a dislike of clothing waste and ‘throwaway culture’	44 (14.8%)	Mixed	Barrier: *“I don’t like to wear something that is repaired."* ID 273 Enabler: *“I don’t like waste. I don’t think fast fashion is good for the planet"* ID 39
Intentions	17 Intentions to repair and repurpose	Interest and willingness to repair and repurpose, including learning new skills	16 (5.4%)	Mixed	Barrier: *“don’t want to do it I guess. Never really crossed my mind."* ID 222 Enabler: *“I have recently purchased a sewing machine and now more interested in what I can do with existing clothing items"*ID 216
Goals	18 Resolve to behave pro-environmentally	Personal goals to consume more sustainably, avoid new purchases and landfill, and reduce one’s environmental footprint	29 (9.8%)	Enabler	*“I pledged not to buy new clothes unless absolutely essential about two and a half years ago and would repair clothes if necessary."* ID 99

**Table 7:** Proposed Intervention Logic Model.

Barrier/Enabler (THF Domain)	Intervention Type(s)	Selected BCTs (See [45] for Descriptions and Numbering System)	BCT Operationalisation	Policy Option(s)
Beliefs about benefits of repair and repurpose (beliefs about consequences) Attachment to clothing (emotion)	Education Persuasion	5.3 Information about social and environmental consequences 5.2 Salience of consequences	Explain environmental, economic, and emotional benefits of repairing and repurposing clothes (less waste, money saved, increased value) Present images of consequences (e.g., textile waste) to highlight environmental threat and create negative feelings about buying new and disposing clothes	Communication/-marketing	
•Incongruence with identity (identity)	•Persuasion	13.5 Identity associated with behaviour change	•Advise to construct a new identity as a sustainable and responsible citizen	•Communication/ -marketing	
Dynamic norms (social influences) Attitudes towards repair and repurpose (beliefs about consequences)	Persuasion	6.3 Information about others’ approval 9.1 Credible source	Present images showing citizens repairing and repurposing in different settings (home, in the park with friends, on public transport) Present videos or images of role models explaining benefits of repair and repurpose	Communication/-marketing	
Lack of skills (skills) Dynamic norms (social influences) Routine of repair and repurpose (reinforcement) Ability to focus (memory, attention, and decision processes)	Training Modelling	6.1 Demonstration of the behaviour 4.1 Instruction on how to perform the behaviour 8.2 Behavioural practice/rehearsal	Use a fashion influencer to demonstrate how to repair and repurpose various clothing items via video tutorials and free workshops Provide online guides advising on how to perform repair and repurpose tasks Prompt citizens to practice their skills whenever they have damaged, poorly fitting, or unwanted clothes	Communication/-marketing Service provision	
Lack of access to resources, poor product design, lack of time, unaffordable professional services, competing behaviours (environmental context and resources)	Environmental restructuring Enablement	12.5 Adding objects to the environment 12.1 Restructuring the physical environment	Place free repair kits at store counters Add labels to clothing to indicate durability and repairability Provide free repair and repurpose services in retail stores Create free and accessible repair cafes or community workshops	Service provision Regulation Fiscal measures

## Data Availability

The data presented in this study are openly available via Open Science Framework at https://osf.io/6huwj/ (accessed on 15 April 2022).
