# Supplementary material for: Extending the Lifetime of Clothing through Repair and Repurpose: An Investigation of Barriers and Enablers in UK Citizens
Source: Sustainability. Author manuscript; Available in PMC 2024 Oct 22. (PMC7616726; doi:10.3390/su141710821)
Supplement: Supplementary Materials [file EMS199298-supplement-Supplementary_Materials.zip › sustainability-1810722-supplementary.pdf]

Supplementary Materials

Table S1. Influences on Clothing Repair and Repurpose Scale.

TDF Domain	Items	Source (Adapted from/Content Based on)
Knowledge	1. I know how to repair and repurpose my clothes.	[14] Diddi & Yan (2019)
	2. I am aware of the environmental benefits of repairing and repurposing my clothes.	[14] Diddi & Yan (2019)
	3. I know where to get my clothes repaired or repurposed via professional services.	[72] Nazli (2021)
Skills	4. I have the physical skills to repair and repurpose my clothes (e.g., dexterity to thread a needle).	[20] Laitala & Klepp (2018)
	5. I have the mental skills to repair and repurpose my clothes (e.g., designing alterations).	[20] Laitala & Klepp (2018)
	6. I have training in clothing repair and repurpose (e.g., craft education).	[7] EAC (2019)
Memory, attention, and decision processes	7. I can focus my attention in order to repair and repurpose my clothes	[27] Twigger Holroyd (2016)
	8. I often forget to repair and repurpose my clothes.*^	Original item
Behavioural regulation	9. I lack self-control when buying new clothes, when I could repair and repurpose my existing clothes instead.*^	Original item
	10. I often put off repairing and repurposing my clothes (e.g., not bothered).	[7] EAC (2019)
Social/professional role and identity	11. Repairing and repurposing things is part of my identity.	[22] Armstrong et al. (2015)
	12. I repair and repurpose my clothes to express my individuality and personal style.	[31] Lapolla & Sanders (2015)
Beliefs about capabilities	13. I am confident in my abilities to repair and repurpose my clothes.	[14] Diddi & Yan (2019)
Optimism	14. I am optimistic that the end result of repairing and repurposing my clothes will be successful.	[64] Laitala et al. (2021)
Beliefs about consequences	15. I believe that repairing and repurposing my clothes has positive impacts on the environment (e.g., less waste).	[14] Diddi & Yan (2019)
	16. I believe that repair and repurpose adds value to my clothes by extending their lifetime.	[22] Armstrong et al. (2015)
Intentions	17. I strongly intend to repair and repurpose my clothes.	[50] Fisher et al. (2008)
Goals	18. A goal of mine is to learn new skills to repair and repurpose my clothes.	[31] Lapolla & Sanders (2015)
	19. A goal of mine is to reduce my clothing purchases and textile waste.	[29] Durrani (2018)
Reinforcement	20. I routinely dispose of my clothes instead of repairing and repurposing them.*	[23] Goworek et al. (2012)
	21. I routinely buy new clothes instead of repairing and repurposing my existing ones.*	[23] Goworek et al. (2012)
	22. I feel immediate gratification from buying new clothes.*	[14] Diddi & Yan (2019)
	23. If I repair and repurpose my clothes, I will feel good about myself for caring for the environment.	[14] Diddi & Yan (2019)
Emotion	24. I repair and repurpose my clothes because I have emotional attachment to them.	[14] Diddi & Yan (2019)
	25. I do not repair and repurpose my clothes because I am bored or discontent with them.*	[14] Diddi & Yan (2019)
	26. To me, repairing and repurposing clothing is relaxing.	[22] Armstrong et al. (2015)
	27. I feel pride or accomplishment from repairing and repurposing my clothes.	[72] Nazli (2021)

	28.	I would feel guilty about not repairing or repurposing my clothes that are still good quality.	[22] Armstrong et al. (2015)
Environmental context and resources	29.	I have the necessary equipment (e.g., sewing machine) to repair and repurpose my clothes.	[50] Fisher et al. (2008)
	30.	There is a lack of accessibility to professional repair services near me.*	[50] Fisher et al. (2008)
	31.	Professional repair services are too expensive.*	[34] McLaren & McLauchlan (2015)
	32.	If brands offered repair services or kits after purchase, I would use this.	[22] Armstrong et al. (2015)
	33.	Low prices and wide availability of new clothing make it less likely that I will repair and repurpose my existing clothing.*	[33] Gwilt (2014)
	34.	I am not likely to repair and repurpose clothing that is of low quality or durability.*	[14] Diddi & Yan (2019)
	35.	I do not have the time to repair and repurpose my clothes.*	[14] Diddi & Yan (2019)
	36.	If my community held repair and repurpose events (e.g., repair cafes), I would attend.	[14] Diddi & Yan (2019)
Social influences	37.	Repairing and repurposing clothing is viewed as the norm in society.^	[25] Harris et al. (2016)
	38.	I would repair and repurpose my clothes if fashion trends didn't change so often*	[7] EAC (2019)
	39.	I feel social pressure to buy new clothing in order to fit in*	[14] Diddi & Yan (2019)
	40.	I do not repair and repurpose my clothes because other people see it negatively.*	[33] Gwilt (2014)

Note. * indicates reverse-coded items; ^ indicates items were removed in final analysis due to low reliabilities.

Table S2. Ordinal Logistic Regression—Clothing Repair and Repurpose Behaviour Before COVID-19.

Predictor	β	SE (β)	Wald's χ^2	df	p	Odds Ratio	[95% CI Odds Ratio]
Knowledge	0.37	0.20	3.21	1	0.07	1.44	[0.97, 2.16]
Skills	0.26	0.21	1.54	1	0.21	1.30	[0.86, 1.96]
Memory, Attention & Decision Processes	0.13	0.14	0.92	1	0.34	1.14	[0.87, 1.50]
Behavioural Regulation	−0.02	0.11	0.04	1	0.83	0.97	[0.79, 1.21]
Social Influences	−0.17	0.16	1.03	1	0.31	0.85	[0.61, 1.17]
Environmental Context & Resources	0.21	0.29	0.54	1	0.47	1.24	[0.70, 2.18]
Identity	0.16	0.15	1.14	1	0.29	1.18	[0.87, 1.59]
Beliefs about Capabilities	0.02	0.17	0.01	1	0.92	1.02	[0.73, 1.42]
Optimism	−0.04	0.15	0.06	1	0.81	0.97	[0.73, 1.28]
Beliefs about Consequences	0.24	0.20	1.44	1	0.23	1.27	[0.86, 1.88]
Intentions	0.16	0.17	0.90	1	0.34	1.18	[0.84, 1.66]
Goals	−0.16	0.15	1.14	1	0.29	0.85	[0.63, 1.15]
Reinforcement	0.76	0.23	10.77	1	0.001	2.13	[1.36, 3.36]
Emotion	0.14	0.24	0.34	1	0.56	1.15	[0.72, 1.86]
**Test**			χ^2	df	p		
Test of parallel lines for proportional odds assumption			41.20	28	0.06		
Omnibus test for model fit (Likelihood Ratio)			169.11	14	<0.001		
Pseudo R^2			0.22 (McFadden R^2)				

Table S3. APEASE Criteria for Intervention Types.

Candidate Intervention Type	Affordability (Yes/No)	Practicability (High/Medium/ Low)	Effectiveness (High/Medium/ Low)	Acceptability (High/Medium/ Low)	Side-effects (Positive/None/ Negative)	Equity (Increase/None/ Decrease)	Justification	Decision (Yes/No)
Education	Yes	High	Medium	High	Positive	Increase	Meets all criteria, evidenced by the 'Love Your Clothes' awareness campaign by WRAP. Evaluation is needed as longer-term effectiveness is not yet clear.	Yes
Persuasion	Yes	High	High	High	Positive	None	Persuasive messaging (words, images) meets all criteria.	Yes
Incentivisation	No	Low	High	Medium	Negative	Decrease	Can have negative side-effects (e.g., bringing clothes to be repaired in-store in return for a voucher might fuel the purchase of new clothes)	No
Coercion	No	Low	High	Low	Negative	Decrease	Not acceptable nor enforceable to create an expectation of punishment or cost if clothing is not repaired or repurposed.	No
Training	Yes	High	Medium	High	Positive	Increase	Meets all criteria, evidenced by 'Habits for Life' online workshops by WRAP. Evaluation is needed as longer-term effectiveness is not yet clear.	Yes
Restriction	No	Low	High	Low	Negative	Decrease	Not acceptable nor practicable to restrict competing behaviours (e.g., buying new and disposing clothes)	No
Environmental restructuring	Yes	Medium	High	High	Positive	Increase	Changes to the micro-environment likely meets all criteria.	Yes
Modelling	Yes	High	Medium	High	None	None	Using fashion influencers as models to imitate likely meets all criteria.	Yes
Enablement	Yes	Medium	High	High	Positive	Increase	Meets all criteria, evidenced by success of repair cafes, and some retailers offering free repair services.	Yes

Table S4. APEASE Criteria for Policy Options.

Candidate Policy Option	Affordability (Yes/No)	Practicability (High/Medium/Low)	Effectiveness (High/Medium/Low)	Acceptability (High/Medium/Low)	Side-effects (Positive/None/Negative)	Equity (Increase/None/Decrease)	Justification	Decision (Yes/No)
Communication/marketing	Yes	High	High	High	None	None	Mass media campaigns (e.g., 'Love Your Clothes' by WRAP) meet all criteria.	Yes
Guidelines	Yes	High	Medium	Medium	None	None	May not be effective over and above regulatory policies.	No
Regulation	Yes	High	High	Medium	None	None	Development and implementation of rules to regulate the fashion industry to support citizens to repair and repurpose meets all criteria.	Yes
Legislation	Yes	Medium	High	Low	Negative	Decrease	Not practicable to implement currently. Regulation (middle ground between guidelines and legislation) is more appropriate and feasible.	No
Environmental/social planning	No	Low	Medium	Medium	None	None	Not affordable to make large-scale changes to the macro-environment.	No
Service provision	Yes	Medium	High	High	Positive	Increase	Meets all criteria, as free repair services and community workshops will improve ability and opportunity to change behaviour. A challenge would be delivering such services so that engagement is high.	Yes
Fiscal measures	Yes	High	High	High	None	Increase	Reducing tax rates to encourage the behaviour meets all criteria.	Yes

Table S5. APEASE Criteria for Selecting BCTs from Most Frequently Used Lists (Selected BCTs in bold).

Intervention type	Most frequently used BCTs	Relevant to context?	Meets APEASE criteria?
Education	**Information about social and environmental consequences**	**Yes**	**Yes, meets all criteria**
	Information about health consequences	No	–
	Feedback on behaviour	Yes	No, not practicable
	Feedback on outcomes of the behaviour	Yes	No, not practicable
	Prompts/cues	Yes	No, not practicable
	Self-monitoring of behaviour	No	–
Persuasion	**Credible source**	**Yes**	**Yes, meets all criteria**
	Information about social and environmental consequences	As above	As above
	Information about health consequences	As above	As above
	Feedback on behaviour	As above	As above
	Feedback on outcomes of the behaviour	As above	As above
Training	**Demonstration of the behaviour**	**Yes**	**Yes, meets all criteria**
	**Instruction on how to perform a behaviour**	**Yes**	**Yes, meets all criteria**
	Feedback on behaviour	As above	As above
	Feedback on outcomes of the behaviour	As above	As above
	Self-monitoring of behaviour	As above	As above
	**Behavioural practice/rehearsal**	**Yes**	**Yes, meets all criteria**
Modelling	Demonstration of the behaviour	As above	As above
Environmental restructuring	**Adding objects to the environment**	**Yes**	**Yes, meets all criteria**
	Prompts/cues	As above	As above
	**Restructuring the physical environment**	**Yes**	**Yes, meets all criteria**
Enablement	Social support (unspecified)	No	–
	Social support (practical)	No	–
	Goal setting (behaviour)	No	
	Goal setting (outcome)	No	
	Adding objects to the environment	As above	As above
	Problem solving	No	–
	Action planning	No	–
	Self-monitoring of behaviour	As above	As above
	Restructuring the physical environment	As above	As above
	Review behaviour goals	No	–
	Review outcome goals	No	–
